# Exploring Periphytic Biofilms as Nature’s Cleanup Crew for Contaminated Surface Waters

**DOI:** 10.1007/s00267-025-02381-1

**Published:** 2026-02-18

**Authors:** Tatiani Andressa Modkovski, Charles Windson Isidoro Haminiuk, Bárbara Alves de Lima Nawate, Juliane Ribeiro das Chaves, Júlio César Rodrigues de Azevedo

**Affiliations:** 1 Departamento de Hidráulica e Sanitária, Universidade Federal do Paraná, Curitiba, Paraná Brasil; 2 Departamento de Química e Biologia, Universidade Tecnológica Federal do Paraná, Curitiba, Paraná Brasil

**Keywords:** Biofilm, Biodegradation, Bioremediation, River, Self-cleaning

## Abstract

Periphytic biofilms, formed by fungi, bacteria, algae, and protozoa within an extracellular matrix, colonize various surfaces in river water and play a key role in carbon and nutrient cycling and river self-purification. Given their ecological importance, understanding the mechanisms these biofilms employ in contaminant bioremediation is essential for optimizing their application in environmental management. To achieve this, it is crucial to differentiate processes such as sorption, bioaccumulation, biodegradation, and biotransformation, which are key to evaluating bioremediation strategies using biofilms. This review highlights the effectiveness of biofilms in contaminant removal, even at low concentrations, due to their extensive adherence to solid surfaces in river systems. Furthermore, it explores the potential mechanisms of biofilm action in bioremediation. The review also addresses current challenges and prospects for enhancing the self-purification of aquatic ecosystems, alongside applying green bioremediation technologies utilizing periphytic biofilms. Such advancements aim to contribute to the sustainable management of water resources and restore aquatic ecosystem health.

## Introduction

The increasingly intense use of pharmaceuticals, personal care products, endocrine disruptors, insecticides, pesticides, metals, and dyes, among numerous other contaminants, underscores the need to investigate their occurrence (Montagner et al., 2019), fate, persistence, impacts, and strategies for their removal (Maia et al., 2023; Mukhopadhyay et al., 2022). These substances, referred to as emerging contaminants (ECs), have been detected globally in surface water, wastewater, and groundwater, where they pose risks to ecosystems, aquatic organisms, and human health (Mishra et al., 2022).

Among the natural processes with potential to mitigate these contaminants, microbial biofilms stand out as the most dominant form of life in streams (Battin et al., 2003). River biofilms are essential components of aquatic ecosystems, composed primarily of algae, bacteria, fungi, microfauna, and EPS (extracellular polymeric substances) that attach to solid surfaces such as gravel, sediments, or aquatic plants (Battin et al., 2016). EPS constitute a complex matrix of biopolymers, including polysaccharides, proteins, lipids, and nucleic acids, that are secreted by microorganisms themselves. This matrix acts as the structural scaffold of the biofilm, providing mechanical stability, mediating adhesion to surfaces, retaining water and nutrients, and protecting cells from environmental stressors such as toxic compounds or fluctuations in flow (Flemming and Wingender, 2010). As a result, biofilms can capture particles and fine sediments that accumulate in the water and play a fundamental role in biogeochemical cycling, chemical exchanges between surface water and the riverbed (Gong et al., 2023).

Biofilms exhibited high concentrations of certain contaminants, emphasizing their strong attraction and interaction with these substances (Wang et al., 2019; Writer et al., 2011). Therefore, considering the biofilms’ ability to sorb contaminants, they can be regarded as pollution indicators and act as sinks for effectively removing contaminants (Shabbir et al., 2017a). Additionally, due to their diverse microbial communities, river biofilms can significantly contribute to the degradation of ECs in aquatic environments, making them essential components in natural attenuation and in situ bioremediation processes (Lu et al., 2014; Nadeau et al., 2021; Sharma et al., 2020; Singh et al., 2020).

Conventional domestic wastewater treatment plants, such as stabilization ponds and activated sludge systems, were not originally designed to handle recalcitrant organic pollutants, including ECs (Morin-Crini et al., 2022). Physicochemical methods and advanced oxidation processes, such as adsorption, membrane filtration, and ozonation, have demonstrated efficiency in contaminant removal. However, they are often limited by high energy requirements and operating costs, and the potential generation of toxic byproducts (Hu et al., 2024; Das et al., 2023). Biological treatments, on the other hand, are more sustainable and cost-effective, but traditional approaches based on suspended microbial cultures may lack stability and efficiency in degrading persistent compounds. In this context, biofilm-based systems represent a novel and advantageous strategy. Structured microbial communities provide greater resilience, adaptability, and multifunctionality than planktonic microorganisms, improving contaminant sorption and degradation (Mishra et al., 2022).

Bioremediation is a cost-effective and environment-friendly treatment technology for biodegrading organic and inorganic pollutants in contaminated areas, utilizing microorganisms’ metabolic capabilities (Padma et al., 2023; Sharma and Kumar, 2021). Indigenous microorganisms have significant potential to degrade contaminants due to their adaptability, genetic diversity, and functionality, removing pollutants through processes such as bioaccumulation, biosorption, biotransformation, and biomineralization (Padma et al., 2023; Tang et al., 2022).

Natural river biofilms hold significant potential for contaminant removal, however their application in bioremediation remains underexplored. Their dynamic and responsive behavior in contaminated environments, which enables rapid adaptation and efficient pollutant removal, warrants further investigation. The novelty of this review lies in synthesizing current knowledge on natural river biofilms as agents for the bioremediation of contaminated water, emphasizing the key mechanisms through which they interact with and eliminate contaminants, as well as the environmental conditions that regulate their functioning. Thus, this work offers new perspectives and practical considerations on the use of natural river biofilms in sustainable water management.

## Methodology

Literature searches were performed using the Web of Science database, with the following broad parameters: Search in “All Databases”; Collections “All”; Documents “Topic” (searches on title, abstract and indexing). The search was limited to peer-reviewed articles published in English and focused on periphytic and river biofilms. The publication period considered ranged from 2014 to 2024. A combination of keywords was used, including “periphytic biofilms”, “river biofilms”, “periphyton”, “bioremediation”, “degradation”, “pollutant removal”, “emerging contaminants”, “metals”, “pharmaceuticals”, “dyes”, and “microplastics”. Boolean operators (AND/OR) were applied to refine the searches and combine related terms.

The initial search yielded approximately 2454 articles. After duplicate removal and screening of titles and abstracts for relevance to the scope of this review, approximately 200 articles were retained for full-text evaluation. Studies were included if they were original research articles and addressed contaminant removal or transformation by periphytic or river/aquatic biofilms under natural or environmentally relevant conditions. Studies focusing exclusively on biofilms formed by single microbial species or biofilms developed in engineered bioreactors were excluded.

Following full-text assessment, a total of 27 studies were selected and synthesized in this review (Table 1). Additional relevant publications were identified through backward and forward citation tracking of key articles to ensure the inclusion of seminal and recent contributions. The selected studies were analyzed qualitatively, emphasizing removal mechanisms, and environmental context, rather than quantitative meta-analysis.

**Table 1:** Studies on the use of periphytic biofilms in the bioremediation of contaminated aquatic environments (2014–2024)

Compound	Initial concentration	Removal mechanisms	Efficiency	References
Cu	0.5 e 2 mg L^−1^	Biosorption and bioaccumulation	99 e 98%	(Zhong et al., 2020)
Cu (II)	2, 5, 10 and 20 mg L^−1^	Biosorption	98, 90, 78 e 78%	(Liu et al., 2018)
Cu	2 e 10 mg L^−1^	-	99%	(Ma et al., 2018)
Glyphosate	<10 μg L^−1^	Degradation	100%	(Carles et al., 2019)
Glyphosate	0.132 mM of P-equivalents	Degradation	100%	(Rossi et al., 2021)
Methomyl	50 mg L^−1^	Degradation	90.6%	(Chen et al., 2015)
Carbofuran	50 mg L^−1^	Degradation	52.5%	(Tien et al., 2017)
Metazachlor, metribuzin and bentazone	540, 62 and 150 μg L^−1^	Biodegradation	> 94%	(Bighiu and Goedkoop, 2021)
Lindane and norfloxacin (NOR)	Lindane (0.2–2.0 μmol L^−1^) and NOR (40–400 μmol L^−1^)	Adsorption	45.6% (Lindane) and 98.9% (NOR)	(Dong et al., 2018)
Ofloxacin	0.1–10 mg L^−1^	Sorption	-	(Zhang et al., 2018)
Phenanthrene and ofloxacin	0.3–6.0 and 0.1–3.0 μmol L^−1^	Sorption	-	(Wang et al., 2019)
Azithromycin (AZI)	5, 50, 200, 500, 5000 μg L^−1^	Biodegradation	88.65, 87.50, 80.62, 54.37 and 44.70%	(Liang et al., 2024)
Venlafaxine, diuron and triclosan	10 μg L^−1^ (diuron and triclosan) and 50 μg L^−1^ (venlafaxine)	Bioaccumulation and biotransformation	15% (Venlafaxine), 6.7% (Diuron) and 6% (Triclosan)	(Santos et al., 2019)
Iopromide (IOP)	100 μg L^−1^	Transformation	93%	(Mauch et al., 2023)
Sulfamethoxazole (SMX) and doxylamine succinate (DOX)	SMX (0, 0.05, 1, 2, and 5 mg L^−1^) and DOX (0, 0.05, 0.1, 0.2, and 1 mg L^−1^)	Bioadsorption, Bioaccumulation and Biodegradation	78.7, 18.9, 1.7% (SMX) and 24.3, 15.9, 58.6% (DOX)	(Yadav et al., 2021)
Erythromycin (ERY) and roxithromycin (ROX)	0.5 μg L^−1^, 5 μg L^−1^ and 50 μg L^−1^	Biodegradation and indirect photodegradation	51.63–66.87% (ERY) and 41.85–48.27% (ROX)	(Yan et al., 2023)
17α-ethinylestradiol (EE2), bisphenol A (BPA) and polypropylene microplastic (PP)	0.2–5 mg L^−1^	Biodegradation	100%, 100%, and 5–12%	(Shabbir et al., 2022)
Polypropylene (PP), polyethylene (PE) and polyethylene terephthalate (PET)	Dimensions pellets < 1000 μm	Biodegradation	18% (PP), 14% (PE) and 19.7% (PET)	(Shabbir et al., 2020)
63 target substances	1 mg L^−1^	Biotransformation and Bioaccumulation	-	(Desiante et al., 2021)
Inorganic phosphorus	13 mg L^−1^	Adsorption	90%	(Lu et al., 2014)
Orange methyl azo dye	25–500 mg L^−1^	Biotransformation and Bioaccumulation	100%	(Shabbir et al., 2017a)
Azo dye amaranth	50–500 mg L^−1^	Bidegradation	100%	(Shabbir et al., 2017b)
Crystal violet dye	25–1000 mg L^−1^	Biotransformation and Bioaccumulation	100%	(Shabbir et al., 2018)
Methylene Blue, Crystal Violet, and Oxytetracycline	10 mg L^−1^	Biosorption and Bioaccumulation	80, 60 and 40%	(Bursztyn Fuentes et al., 2024)
Nitrogen and phosphorous	Total nitrogen = 4.5 mg L^−1^ and total phosphorous = 2.0 mg L^−1^	-	< 2.0 and 0.02 mg L^−1^,	(Liu et al., 2016)
Cyanobacterial bloom	-	Growth inhibition	Microcystin < 1 μg L⁻¹	(Ko et al., 2019)
Cyanobacterial bloom	-	Growth inhibition	Microcystis 70.8% and Dolichospermum 94.8%,	(Van Le., 2023)

## Diversity and Function of Periphytic Biofilms

**Fig. 1:**
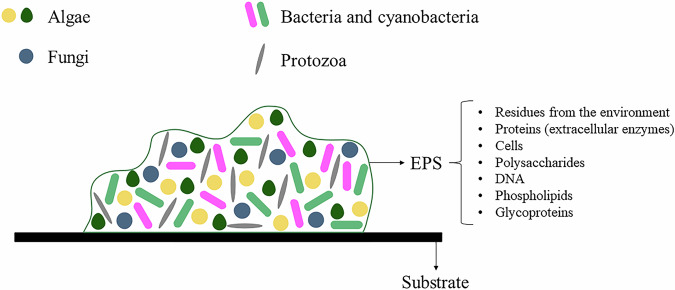
Physical structure of periphytic biofilms with their main components. DNA (Deoxyribonucleic Acid), EPS (extracellular polymeric substances)

Microorganisms are the most prevalent life forms on Earth, and approximately 99.9% of microbial biomass in subsurface environments adheres to surfaces in the form of biofilm (Su et al., 2023). Biofilms are complex and dynamic structures as they are constantly exposed to changes in aquatic ecosystems. Periphyton is a biofilm that grows on submerged surfaces in rivers, lakes, swamps, marshes, beaches, rice fields, and other locations (Tang et al., 2023). As shown in Fig. 1, they are composed of microbial cells, which can be eukaryotic or prokaryotic, adhered to an inert surface (Fernandes et al., 2020). The interaction of these microorganisms with various interfaces includes connections between solid and liquid phases, liquid and liquid phases, liquid and gas phases, solid and gas phases, and even interactions among the microorganisms themselves in the form of aggregates (Sentenac et al., 2022).

The terminology used for biofilms may vary according to the surface to which they adhere. When attached to rocks, they are referred to as “epilithic biofilm” or “epilithon”; on wood, as “pixylic biofilm”; on sand grains, as “episammon”; on muddy sediments, as “epipelon”; on sediments, as “metaphyton”; and on plant organisms, as “epiphyton.” All these terminologies fall under the broader category of “periphyton” or “periphytic biofilms,” which constitute river biofilms. In addition to these natural substrates, periphytic biofilms also grow on artificial substrates, such as plastics, glass, and other materials found in aquatic environments (Sentenac et al., 2022).

In addition to microbial cells, periphytic biofilms contain EPS and water. The EPS matrix comes from residues from the environment and the metabolism of these microorganisms. Water is the most significant fraction (97%) of the biofilm matrix (Fernandes et al., 2020). Furthermore, the composition of EPS and water provides a hydrated environment that takes longer to dry than the surrounding environment and protects the biofilm cells from fluctuations (Flemming and Wingender, 2010).

The part composed of EPS acts as a protection for microorganisms and as a structure that promotes the binding of all fractions within the biofilm. This fraction contains proteins (extracellular enzymes), cells, polysaccharides, DNA (Deoxyribonucleic Acid), phospholipids, and glycoproteins (Fig. 1). The EPS of a biofilm varies from one location to another, as it depends on the microorganisms present there, the available nutrients, and other physical parameters, such as the shear forces suffered, pH, and temperature (Fernandes et al., 2020; Flemming and Wingender, 2010).

Certain microorganisms in biofilms can also survive in planktonic communities or move from biofilms to planktonic communities and vice versa. These microorganisms in biofilms can persist in the environment due to the surrounding EPS matrix. Conversely, when they are in planktonic form, they can disperse and establish themselves in new environments (Sentenac et al., 2022). It is important to emphasize that bacteria dominate the microorganisms present due to their fast reproduction and adaptability to many conditions (Fernandes et al., 2020).

The biodegradation conducted by biofilms represents better real-world degradation scenarios compared to planktonic assays. This is because most environmental biodegradation events occur through biofilm-substrate interactions (Nadeau et al., 2021). Biofilms provide information on the spatial and temporal aspects of degradation, including the associations between degradation and biofilm adhesion and growth, chemical movement, mobility, and cell characteristics. Additionally, biofilm assays offer insights into how different species interact, wich helps to identify synergistic or competitive relationships that can enhance or inhibit biodegradation processes (Nadeau et al., 2021).

Biofilms play a significant role in river ecosystems, driving enzymatic activities and contributing to critical processes such as biogeochemical cycling, organic matter cycles, primary production, and ecosystem respiration. They also facilitate the natural purification of surface waters and stabilize sediments, thereby mitigating erosion. These functions highlight the role of biofilms in maintaining the ecological balance and health of riverine environments (Gerbersdorf and Wieprecht, 2015; Dömölki et al., 2022).

A hallmark of biofilms is the formation of a three-dimensional extracellular matrix that sustains prolonged microbial juxtaposition and close cell–cell interactions (Flemming et al., 2016). This architecture confers enhanced structural organization and functional productivity relative to equivalent planktonic assemblages (Hansen et al., 2007). Consequently, biofilms exhibit emergent system-level properties, such as elevated horizontal gene transfer, particularly of antimicrobial resistance determinants, along with active sorption dynamics, efficient transport via pores, voids, and channel networks, and the extracellular sequestration of water, nutrients, and enzymes (Sentenac et al., 2022). Notably, cooperative interactions between cells, mediated by chemical and electrical communication, may represent the most striking characteristic of biofilms, revealing evidence of coordinated behavior and division of labor typically associated with multicellular organisms (Flemming and Wingender, 2010; Flemming et al., 2016; Hansen et al., 2007).

Collectively, these structural and functional attributes underpin the capacity of biofilms to retain and accumulate dissolved substances from their surrounding environment. As a result, biofilms are regarded as effective bioaccumulators of nutrients and microcontaminants, including pesticides, pharmaceuticals, metals, and microplastics (Fernandes et al., 2020). Nevertheless, the development of water treatment strategies based on biofilms, whether in situ or ex situ, requires a deeper understanding and evaluation of contaminant removal processes, particularly in periphytic biofilms.

## Possible Mechanisms of Action of Biofilms in Bioremediation

Biofilm microorganisms in aquatic environments face challenging and selective conditions, leading to structural and metabolic adaptations that enable their survival. This adaptive strategy results in varied responses to contaminants, including degradation and biotransformation (Desiante et al., 2021). In this way, biofilms also play a role in the bioremediation and self-purification of the environments they inhabit.

Biofilm-mediated bioremediation of environmental contaminants utilizes both cells and EPS (Mahto et al., 2022). In the biofilm and matrix-EPS, different exoenzymes degrade various xenobiotics, charged groups trap organic contaminants via biosorption, and bacteria use these toxic contaminants as a carbon and energy source for their growth and development. Hence, natural biofilms can eliminate microcontaminants from aquatic environments, functioning as a natural repository through passive sorption or engaging in various biological processes such as biosorption, bioaccumulation, biodegradation, or biotransformation (Desiante et al., 2021).

**Fig. 2:**
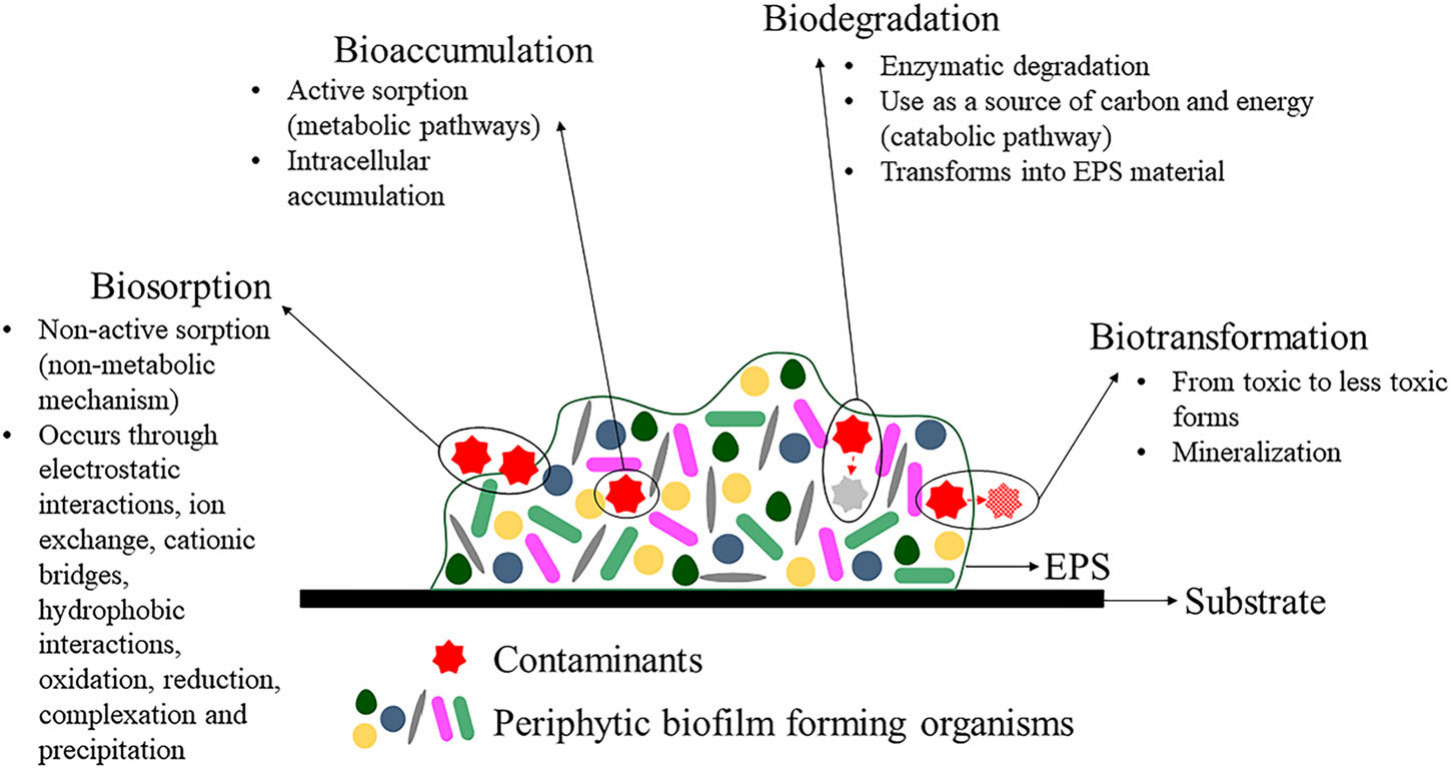
Schematic diagram demonstrating the possible mechanisms used by periphytic biofilms in the bioremediation of contaminants

The mechanisms of action involved in the bioremediation of contaminants by biofilms are fundamental to understanding this process, but distinguishing between biodegradation, biotransformation, sorption, and bioaccumulation is challenging. Figure 2 summarizes the mechanisms employed by periphytic biofilms in the bioremediation of various contaminants. This figure clarifies the metabolic and physical pathways involved in each mechanism, offering a detailed view of the role of each in the pollutant removal process and contributing to a better understanding of the dynamics of bioremediation.

### Biosorption and Bioaccumulation

Biosorption and bioaccumulation are fundamental mechanisms by which periphytic biofilms contribute to the remediation of contaminants in aquatic environments. Sorption is a process involving both adsorption and absorption, and it can be either active or passive. When sorption is active, it involves metabolic pathways and is referred to as bioaccumulation (Chan et al., 2022). On the other hand, biosorption is a passive, non-metabolic mechanism without energy consumption, which results from the surface sorption of contaminants by microorganisms and EPS in periphyton (Liu et al., 2017). While biosorption can occur in living and dead cells, bioaccumulation is exclusive to living cells that metabolize the absorbed substances for growth and development (Bhunia et al., 2022). As illustrated in Fig. 2, in biosorption, contaminants are retained on the external surface of the biofilm, whereas in bioaccumulation, contaminants are internalized within the biofilm matrix through active cellular processes.

Periphytic biofilms, due to the complex structure of their EPS matrix, are highly effective at sorbing a wide range of contaminants, including both polar and non-polar compounds. Non-polar contaminants, such as hydrophobic organic pollutants, are primarily adsorbed based on their octanol-water partition coefficient (log Kow), with higher log Kow values indicating greater sorption affinity. Conversely, polar contaminants are sorbed through electrostatic interactions, cation exchange, hydrophobic interactions, and cation bridging. These passive sorption mechanisms are mediated by functional groups located on the surface of EPS, cell membranes, and cell walls (Fernandes et al., 2020). The non-specific nature of biofilm sorption allows for the accumulation of essential nutrients and toxic substances, which is crucial for biofilms’ environmental monitoring and remediation.

Regarding metal remediation, bioaccumulation involves the active uptake of metal ions into the microbial cytoplasm, utilizing metabolic processes that convert these ions into less toxic forms. In contrast, passive biosorption of metals occurs through electrostatic interactions between positively charged metal ions and negatively charged functional groups on the biofilm surface. These interactions facilitate complexation, precipitation, and ion exchange, allowing biofilms to immobilize and detoxify metals without direct metabolic involvement (Mahto et al., 2022; Wu, 2017).

In 2024, Ji and Zhao published a review on the relation between biofilms and Perfluoroalkyl and poly-fluoroalkyl substances (PFASs), also known as “forever chemicals.” According to the authors, biofilms have rough surfaces that contain complex functional groups capable of adsorbing and retaining PFASs in water through known and unknown mechanisms. Studies show that long-chain PFASs containing more than seven fluorinated carbons are more likely to accumulate in biofilms than short-chain PFASs. However, further studies are required to investigate the mechanisms involved in the adsorption of PFASs in biofilms, as both long-chain and short-chain PFASs are widely present in the aquatic environment.

Furthermore, biofilms colonizing substrates like microplastics significantly enhance the retention of contaminants. For instance, Bhagwat et al. (2021) demonstrated that microplastic fibers coated with biofilms accumulated 75 times more perfluorooctane sulfonate (PFOS) than those without biofilm coverage. Functional groups such as carboxyl and sulfonic acid on biofilm surfaces facilitate this, aiding in the hydrophobic and electrostatic capture of the contaminants (Ji and Zhao, 2024).

Microorganisms within periphytic biofilms can utilize the organic contaminants they adsorb as nutrient sources, transforming them into cellular material like cytoplasm and promoting the production of EPS. This capacity for both sorption and subsequent biodegradation of organic pollutants highlights the dual role of biofilms in contaminant removal (Wu, 2017). For example, Fernandes et al. (2019) reported that epilithic biofilms are capable of both adsorbing and biodegrading the herbicide glyphosate, suggesting that the combination of biosorption and biodegradation can effectively eliminate such pollutants from aquatic environments.

### Biodegradation and Biotransformation

Biodegradation encompasses a range of processes through which microorganisms transform or break down substances in the environment. The substrate may be completely mineralized or only partially transformed (biotransformation), generating metabolites that cannot be further utilized and may accumulate in the medium. In some cases, these metabolites bind to matrix components, such as humic acids, becoming immobilized. Although the original contaminant is removed in all these scenarios, partially degraded products can be more problematic, particularly when the goal is to remediate contaminated environments (Kiel and Engesser, 2015).

Biotransformation is the alteration of compounds by an organism, resulting in changes to its chemical and toxicological properties. It also encompasses the metabolic alterations that occur in organisms due to exposure to xenobiotics (Valentová, 2023). Microorganisms in biofilms have catabolic enzymes that degrade persistent organic pollutants. In addition, microorganisms biotransform and mineralize different toxic organic pollutants (Mahto et al., 2022). These processes can occur in the presence or absence of oxygen. For example, anaerobic catabolism uses reductive reactions to degenerate the aromatic ring of hydrocarbons (Ghosal et al., 2016). On the other hand, the aerobic metabolism process uses monooxygenase and dioxygenase enzymes to incorporate oxygen atoms into hydrocarbons, leading to the hydroxylation of the aromatic ring (Mahto et al., 2022). In this context, molecular oxygen acts not only as the terminal electron acceptor but also as a cosubstrate directly involved in hydroxylation reactions and oxygenolytic ring-opening processes (Ghosal et al., 2016). When bacteria use the catabolism pathway of organic pollutants, metabolic end products are formed that enter the Krebs cycle, leading to the formation of CO_2_, water, and energy for the growth and maintenance of microorganism cells (Mahto et al., 2022).

Biodegradation is crucial for removing contaminants and transforming environmental substances (Bose et al., 2021). Carles et al. (2019) performed a study on periphytic biofilm, which showed that biofilms can biodegrade glyphosate in conditions similar to the environment. The experiment did not detect any glyphosate and the metabolite AMPA at the end of the study. The presence of phosphorus was also evaluated, and the best results were for the lowest initial phosphorus concentration. This is because microorganisms in the biofilm use glyphosate as a source of phosphorus. The natural biofilm uses catabolism to degrade glyphosate and obtain energy. Also, according to Carles and Artigas (2020), microorganisms that use glyphosate as a source of phosphorus have an enzymatic complex that allows an orthophosphate molecule to be released from glyphosate or AMPA through biodegradation pathways.

Some authors have stated that biofilm microorganisms require extra carbon sources to degrade certain compounds effectively (Shabbir et al., 2020; Wang et al., 2019). For instance, when glucose is added as a carbon source for microorganisms to obtain energy, the biofilm’s ability to break down the insecticide methomyl carbamate is improved (Chen et al., 2015). In contrast, in petroleum biodegradation, bacteria use polycyclic aromatic hydrocarbons (PAH) as a carbon source and degrade them through several catabolic pathways. Biofilms use emulsification, solubilization, and biodegradation mechanisms to remove petroleum hydrocarbons. The biofilm’s emulsification breaks down large oil droplets into smaller ones, while EPS increases hydrocarbon dispersion and solubility, making biodegradation more efficient (Mahto et al., 2022).

The removal of textile dyes combines adsorption and biodegradation through natural biofilms. According to Shabbir et al. (2017a), dyes are first adsorbed, and biodegradation occurs once adsorption sites are saturated. Biofilms enhance this process due to their large surface area, active sites, and transport pathways. Dye biodegradation involves azo bond cleavage, desulfonation, oxidation, reduction, breakdown into open-chain hydrocarbons, and deamination, with enzymes such as azo reductase, peroxidases, and laccases playing key roles (Shabbir et al., 2017a).

Liang et al. (2024) identified 15 enzymes highly correlated with the biotransformation of azithromycin, energy supply, and antibiotic resistance processes. Key enzymes mentioned include aryl-alcohol dehydrogenases, hydroxylamine dehydrogenase, and monooxygenases, which are directly involved in azithromycin degradation. The study emphasizes that these enzymes facilitate the breakdown of the antibiotic, allowing the microbial community to adapt and enhance its metabolic pathways for more efficient biodegradation. This enzymatic activity is crucial for the overall health of the periphyton community and its ability to mitigate the impacts of antibiotic contamination in aquatic environments.

Mauch et al. (2023) identify key biochemical pathways involved in the biotransformation of iopromide (IOP) by periphyton and microbial communities. It highlights the oxidation of hydroxy groups, cleavage of amide-methylene bonds, and oxidative decarboxylation as primary mechanisms. Co-oxidation, driven by ammonia mono-oxygenase from ammonia-oxidizing bacteria, also plays a significant role in the process, especially under aerobic conditions. These pathways demonstrate the complex interactions between microbial metabolism and environmental factors in the degradation of IOP in aquatic ecosystems.

Biotransformation is related to the enzymatic activities of aquatic microbial communities, and evaluating them is important to improve the degradation of micropollutants in river remediation. To better propose contaminants biotransformation strategies, it is necessary to know the enzymes involved in this process and this is a lack of knowledge in this area. Mass balance calculations and the stoichiometry of compounds help when wanting to prove the biotransformation mechanism. However, to deeply understand and elucidate the biotransformation of contaminants by biofilms, it is necessary to explore the molecular mechanism that occurs (Liang et al., 2024).

Summarizing both processes, as described by Birolli et al. (2019), biodegradation takes place within a biotransformation process when various reactions in the substrate lead to the formation of small molecules and mineralization into water and CO₂. The boundaries between these phenomena are not well defined, but their main distinction lies in the study’s focus and the extent of substrate modification.

## Removal of Contaminants by Periphytic Biofilms

Bioremediation involves detoxifying polluted environments through the action of microorganisms or their byproducts. This process can facilitate the transformation of contaminants into less harmful or inert compounds, such as CO_2_ and water, which pose minimal risk to humans and the environment (Hidalgo et al., 2024). Periphytic biofilms can absorb or biodegrade nutrients or contaminants in their matrix, detoxifying the surrounding environment. Several compounds, such as persistent organic pollutants, pesticides, heavy metals, human and veterinary drugs, nanomaterials, and plastics, can be captured and metabolized in natural biofilms (Sentenac et al., 2022).

Table 1 summarizes studies conducted between 2014 and 2024 exploring using periphytic biofilms as a bioremediation method for removing contaminants in aquatic environments. The table details the evaluated compounds, their initial concentrations in the studies, and the removal mechanisms employed by the microorganisms present in the biofilms.

Zhong et al. (2020) demonstrated that periphyton efficiently biosorb copper (Cu²⁺) from wastewater, reducing Cu concentrations across varying exposure levels. The removal efficiency increased significantly within the first 48 h of treatment, reaching approximately 86% for exposure to 0.5 mg L^−1^ Cu²⁺ and 91% for 2 mg L^−1^. The efficiency continued to rise over time, with final removal rates of about 99% and 98% after 108 h. Although exposure to 2 mg L^−1^ Cu²⁺ inhibited total chlorophyll-a content, the metabolic activity of heterotrophic microorganisms and the rates of chemical oxygen demand (COD) removal remained unaffected.

In addition to these findings, Ma et al. (2018) also reported promising results when investigating periphytic biofilms under similar conditions. In their study, periphytic biofilms demonstrated efficiency, achieving up to 99% Cu removal, even at higher concentrations (2 to 10 mg L^−1^). Additionally, the biofilms adapted to Cu stress by regulating their microbial community composition, which included diverse species such as diatoms and green algae, enhancing their overall capacity for heavy metal removal from wastewater.

Liu et al. (2018) tested immobilizing the biofilm to enhance Cu removal. The authors immobilized the biofilm onto fibers to develop a novel bioreactor. The results indicate that periphyton effectively captures Cu at initial concentrations ranging from 2 to 20 mg L⁻¹, primarily due to the overproduction of EPS and the porous structure of the periphyton, with biosorption serving as the primary removal mechanism.

According to Table 1, the pesticide group is the second largest group of studies evaluating bioremediation in aquatic environments using biofilms. Biofilms contribute to the biodegradation of glyphosate in aquatic ecosystems through their microbial diversity, which enables a range of metabolic pathways for pollutant degradation. They can effectively break down glyphosate, utilizing it as a phosphorus source, which is significant given that glyphosate contains phosphorus (Carles et al., 2019). A study investigated the biodegradation potential of five bacterial strains isolated from glyphosate-contaminated streams, focusing on their ability to metabolize glyphosate and its byproduct, AMPA, as phosphorus sources (Rossi et al., 2021). Both studies demonstrated 100% glyphosate removal under the evaluated conditions.

Periphytic biofilms have also shown potential in the biodegradation of other pesticides, such as methomyl. Bacterial consortia isolated and acclimatized from biofilms removed up to 91% of methomyl within 7 days. Additionally, exposure to methomyl altered the biofilm community structure, favoring species tolerant to the pesticide, which may enhance the overall degradation efficiency (Chen et al., 2015). Bacteria that are resistant to or capable of degrading methomyl produce enzymes that break down the pesticide, metabolize it as a carbon source, or employ mechanisms that prevent it from interfering with their cellular functions. As a result, these bacteria are better equipped to survive and prosper in the presence of methomyl, making them more effective at its degradation (Perpetuini et al., 2023).

Tien et al. (2017) isolated microbial consortia from natural river biofilms by targeting areas that may have been influenced by agricultural activities, which often involve pesticides like carbofuran. The biofilms were then acclimatized and cultured in a laboratory to study their carbofuran degradation abilities under controlled conditions. They achieved a removal rate of 52.5% after 7 days of exposure, compared to only 4.9% removal in control groups without biofilms. The research identified specific bacterial strains, including *Sphingobacterium multivorum*, as effective carbofuran degraders.

Bighiu and Goedkoop (2021) outline future research directions, including identifying specific microbial communities and the genes involved in the biodegradation process. The authors emphasize the importance of conducting field studies to assess degradation rates in natural environments and suggest testing a broader range of herbicides to evaluate degradation patterns across different chemical classes. Finally, implementing long-term monitoring programs is essential to understanding the dynamics of herbicide runoff and leaching from agricultural soils. These efforts aim to enhance our understanding of the ecological roles of biofilms in managing herbicide pollution in aquatic ecosystems.

According Table 1, pharmaceuticals are the most frequently cited ECs in studies on bioremediation of aquatic environments using periphytic biofilms. Natural biofilms exhibit affinity for organic contaminants, including steroidal hormones, lindane, norfloxacin, ofloxacin, phenanthrene, azithromycin, venlafaxine, iopromide, sulfamethoxazole, doxylamine succinate, erythromycin, and roxithromycin (Dong et al., 2018; Liang et al., 2024; Mauch et al., 2023; Santos et al., 2019; Wang et al., 2019; Yadav et al., 2021; Yan et al., 2023; Zhang et al., 2018).

Erythromycin and roxithromycin were degraded by periphyton, with removal rates of 51.63–66.87% and 41.85–48.27%, respectively. Side chain and ring cleavage were identified as the main degradation pathways. Furthermore, while biofilms degrade these antibiotics at high concentrations, the antibiotics also alter river periphyton’s structural composition and photosynthetic processes (Yan et al., 2023). Azithromycin was removed by river biofilms, with removal rates ranging from 44.70% to 88.65% on the 14th day, through three main processes: biosorption, bioaccumulation, and biodegradation. The authors also observed genetic modifications in the biofilm following antibiotic exposure, indicating that biofilms undergo adaptive changes to be resilient to this environmental stress (Liang et al., 2024).

Zhang et al. (2018) and Wang et al. (2019) investigated the adsorption kinetics of pharmaceutical compounds onto biofilms under different conditions. In the study by Zhang et al. (2018), the authors aimed to evaluate the role of extracellular polymeric substances (EPS) in contaminant sorption and concluded that EPS had an inhibitory effect on ofloxacin sorption by natural biofilms. Wang et al. (2019) examined the effects of organic carbon fractions in freshwater biofilms on the sorption of polycyclic aromatic hydrocarbons (PAHs) and antibiotics. Their results showed that biofilm organic carbon fractions influenced the sorption of phenanthrene and ofloxacin, with aliphatic carbon content and surface polarity enhancing sorption. These findings contribute to a better understanding of the significant role of biofilms in the environmental behavior and fate of trace organic contaminants in natural aquatic systems. A detailed understanding of the is essential to reliably assess their role in biogeochemical contaminant cycling.

In addition to the inherent properties of biofilms that can influence sorption and degradation processes, it is also important to more carefully evaluate the partitioning of organic micropollutants and their transformation products in test systems. This evaluation helps to better understand possible pathways of bioaccumulation and biodegradation, as well as the effects of coexisting micropollutants. In this context, river biofilms are a useful model for assessing the effects of both individual and combined exposure to organic micropollutants, as they allow the investigation of responses at the community level (Santos et al., 2019).

Although these studies assess the removal capacity of pharmaceuticals by biofilms, the removal efficiency is generally lower compared to other classes of contaminants (see Table 1). Dong et al. (2018) investigated the sorption of lindane and norfloxacin in biofilms in the presence of heavy metals, such as lead (Pb), cadmium (Cd), chromium (Cr), and arsenic (As). The authors attributed the reduced sorption of lindane in the presence of heavy metals to competitive interactions for sorption sites on biofilm surfaces. In contrast, the presence of metals enhanced the sorption capacity of biofilms for norfloxacin. These findings indicate that metals can selectively modify the sorption mechanisms of different pharmaceuticals, highlighting the complexity of multicomponent interactions in biofilms and suggesting that studies focusing on single contaminants may not adequately represent environmentally relevant conditions.

The prevalence and environmental impact of microplastics, which are among the most widely found pollutants in aquatic environments and marine organisms, necessitates the development of effective removal technologies. Shabbir et al. (2020) demonstrated that periphytic biofilm biodegrades microplastics, with degradation rates of 9.52%–18.02% for polypropylene (PP), 5.95–14.02% for polyethylene (PE), and 13.24–19.72% for polyethylene terephthalate (PET). The biodegradation has been confirmed by microplastic weight loss, Scanning Electron Microscopy (SEM) and Fourier transform infrared spectroscopy (FTIR) analyses. These biofilms are resilient in complex environments and don’t require sterile conditions, making them a cost-effective solution for wastewater treatment. The presence of different carbon sources, especially glucose, enhanced biodegradation efficiency, and altered microbial community structure.

Another study demonstrated an innovative approach for the simultaneous removal of microplastics and endocrine-disrupting chemicals using periphytic biofilms. This capability had not been previously emphasized in studies focused on individual pollutant removal. The study employed four types of periphytic biofilms to remove 17α-ethinylestradiol (EE2), bisphenol A (BPA), and polypropylene (PP). EE2 and BPA (0.2 mg/L each) were completely (~100%) removed within 36 days. Advanced analytical techniques, including Ultra-high performance liquid chromatography (UHPLC), and Gas chromatography coupled with tandem mass spectrometry (GC-MS/MS), were used to evaluate the biodegradation processes and mechanisms. Gel permeation chromatography (GPC) and SEM further validated the biodegradation of PP. Additionally, the study highlights significant changes in microbial community dynamics, particularly an increase in *Proteobacteria* with adding humic acid, which may enhance biodegradation efficiency (Shabbir et al., 2022). This study highlights the potential of periphytic biofilms for the simultaneous removal of pollutants with different chemical properties, offering a promising method for wastewater treatment applications.

Addressing contaminant removal under complex exposure scenarios, Desiante et al. (2021) evaluated the biodegradation and biotransformation of 63 compounds simultaneously using natural biofilms. The selected compounds were based on their frequent occurrence in treated wastewater and surface waters, ensuring environmental relevance, and represented a wide range of use categories, including pesticides, pharmaceuticals, artificial sweeteners, and other anthropogenic substances. In addition, the compounds were chosen to encompass a broad spectrum of functional groups and potential initial biotransformation pathways, such as primary, secondary, and tertiary amides and amines, carbamates, carboxylic acids, chloroacetanilides, esters, nitriles, sulfonamides, triazoles, and halogenated compounds.

The study demonstrated that natural stream biofilms exhibit a high inherent capacity for micropollutant biotransformation, with 31 of the 63 investigated compounds undergoing biotransformation. However, several compounds remained persistent, as 13 substances showed removal rates below 20%. The authors also identified compounds that were removed predominantly through sorption to the biofilms rather than through biotransformation (Desiante et al., 2021). Overall, while biofilms proved effective in removing a substantial portion of the tested micropollutants, removal efficiency varied among compounds, highlighting differences in persistence and removal pathways.

Dyes have also been identified as important contaminants in aquatic environments, and periphytic biofilms have shown significant potential for their removal. Shabbir et al. (2018) reported that periphyton bioreactors completely removed crystal violet (1000 mg L⁻¹) within 144–168 h, with removal rates directly proportional to biomass concentration. Immobilized periphyton also showed strong biodegradation capacity, as minimal desorption occurred after complete decolorization. In a previous study, the same group achieved full decolorization of the azo dye amaranth within 20 h under static conditions, while agitation slowed the process, indicating that static environments favor degradation. Overall, immobilized periphyton demonstrated high potential for dye removal, especially under optimized conditions of pH 7 and 30 °C (Shabbir et al., 2017b).

Biofilms demonstrated effectiveness in the removal of nutrients such as phosphorus and nitrogen from aquatic systems. A study conducted in a eutrophic mixohaline lake in Ushuaia (Argentina) showed that periphyton cultivated on artificial substrates was able to bioaccumulate significant amounts of nitrogen and phosphorus, highlighting its potential as a simple and environmentally friendly remediation strategy. Beyond nutrient removal, the harvested periphytic biomass was successfully reused for the biosorption of organic contaminants, including dyes and an emerging pollutant. Removal efficiencies exceeded 80% for methylene blue and 60% for crystal violet, while nearly 40% removal was achieved for oxytetracycline (Bursztyn Fuentes et al., 2024). The combined capacity of periphyton to remove nutrients during growth and subsequently act as a biosorbent for organic pollutants underscores its dual functionality and aligns well with circular economy principles, in which biomass is not discarded after remediation but repurposed to enhance overall treatment efficiency.

In addition to the examples of contaminant removal by biofilms, periphytic biofilms can also be applied to control the proliferation of bloom-forming cyanobacteria in aquatic systems. In this context, the control does not target cyanobacteria as a functional group within periphyton communities, but rather planktonic, toxin-producing cyanobacteria that form harmful algal blooms, such as *Microcystis*. Periphytic biofilms compete with these planktonic cyanobacteria for dissolved nutrients, particularly nitrogen and phosphorus, thereby limiting their growth in the water column (Ko et al., 2019). Harmful cyanobacterial blooms represent a global environmental concern due to their negative impacts on ecosystem functioning and human health. The cultivation and harvesting of periphyton, composed of a diverse assemblage of heterotrophic and photoautotrophic microorganisms, can reduce nutrient availability and suppress the dominance of bloom-forming cyanobacteria.

To explore strategies with potential for large-scale application in the management of cyanobacterial blooms, Van Le et al. (2023) investigated the response of bacterial communities to periphyton in an outdoor mesocosm environment (1000 L) using high-throughput 16S rRNA gene sequencing data. The study found that periphyton reduced the concentration of planktonic chlorophyll-a by 38.6% and decreased the cell density of *Microcystis* and *Dolichospermum* by 70.8% and 94.8%, respectively. The authors showed that periphyton-modified bacterial interactions favored the growth of organic matter–degrading bacteria, likely enhancing nutrient recycling within the periphyton matrix and reducing nutrient availability in the water column, thereby suppressing cyanobacterial blooms.

Similarly, Liu et al. (2016) evaluated the use of periphyton in combination with a planted floating treatment bed (FTB) for contaminant removal in river systems. Although interactions between periphyton and macrophytes affected root development and light availability, the integrated FTB–periphyton system effectively removed nitrogen and phosphorus from the water. These findings demonstrate that periphyton-based systems, either alone or combined with engineered treatment structures, hold strong potential for upscaling and practical application in the remediation of nutrient-enriched and polluted rivers.

## Mass Balance and Stoichiometry

Stoichiometric and microbial energy reactions occur in biofilm-based bioremediation, where multi-species communities interact. During this process, pollutants are removed from the water and assimilated into the microbial biomass. These chemical reactions are predominantly driven by microorganisms, aiming to capture energy for cellular synthesis and maintain metabolic activity (Wu, 2017). Therefore, studying and describing these processes’ stoichiometry and mass balance is important.

The mass balance of compounds in a bioremediation process is crucial for distinguishing bioaccumulation from biodegradation and biotransformation. Desiante et al. (2021) emphasize that a complete mass balance provides insight into the active bioaccumulation of contaminants in biofilms, enabling the assessment of their role in detoxifying surface waters from foreign substances. The authors aimed to study the fate of complex mixtures of micropollutants at environmentally relevant concentrations in biofilms, specifically focusing on differentiating between biotransformation, passive sorption, and active bioaccumulation. Through mass balance, the amount of contaminant present in the liquid phase represents what was not removed by the biofilm. The amount present in the biofilm at the end of the test was bioaccumulated, and these two concentrations, subtracted from the initial concentration added, represent biotransformation. Tests and mass balance calculations were performed with live and dead biofilm biomass to differentiate active bioaccumulation from passive sorption.

Santos et al. (2019) evaluated the mass distribution of selected organic microcontaminants between water and biofilm in mesocosms. The concentrations obtained for each microcontaminant after 72 h of exposure, as well as the volume of water and the mass of biofilm, were used for the mass balance. The authors used the following equations:

1 $${\rm{Contaminant\; remaining\; in\; water}}( \% )=\frac{{C}_{w}\left(72\mathrm{h}\right)\times \mathrm{V}}{{C}_{w}\left(0\mathrm{h}\right)\times \mathrm{V}}\times 100$$

2 $${\rm{Bioaccumulation}}( \% )=\frac{{C}_{{biofilm}}\left(72\mathrm{h}\right)\times {m}_{{biofilm}}}{{C}_{w}\left(0\mathrm{h}\right)\times \mathrm{V}}\times 100$$

3 $${\rm{Biotransformation}}( \% )=\frac{\sum {C}_{w}{\mathrm{TP}}\left(72{\mathrm{h}}\right)\times \sum {C}_{{biofilm}}{\mathrm{TP}}\left(72{\mathrm{h}}\right)\times\,{m}_{{biofilm}}}{{C}_{w}\left(0\mathrm{h}\right)\times \mathrm{V}}\times 100$$

where C_w_ (μg L^−1^) is the concentration of the corresponding microcontaminant in water at time 0 h or 72 h, V (L) corresponds to the volume of water used in the exposure experiment, C_biofilm_ (μg g^−1^) is the concentration of the corresponding microcontaminant in biofilm, m_biofilm_ (g) corresponds to the mass of biofilm (in dry weight), Σ C_w_ TP (μg L^−1^) is the sum of the concentration of all TPs (transformation products) in water at 72 h and, Σ C_biofilm_ TP (μg g^-1^) is the sum of concentration of all TPs in biofilm at 72 h.

Biotransformation was assessed by considering the total amount of transformation products (TPs) generated, taking into account their occurrence in both water and biofilm. In this context, all possible types of transformations (e.g., biotransformation, phototransformation, etc.) that may take place within the mesocosm during the exposure experiments were included (Santos et al., 2019).

Liu et al. (2023) conducted a study on the degradation of phenol and formaldehyde using the fungus *Aspergillus nomius* SGFA1. The study involved analyzing the biodegradation and biomass accumulation of phenol and formaldehyde-based on the dry weight of the biomass. Mass balance and stoichiometric analysis showed that 0.26 g g^−1^ and 0.05 g g^−1^ of carbon from phenol and formaldehyde were converted into biomass. The remaining carbon was converted into H_2_O and CO_2_. In this particular case, the conversion rate of biomass to phenol was higher than formaldehyde’s. This demonstrates the ability of the microorganism to effectively remove phenol and formaldehyde simultaneously, providing new information on the use of microorganisms to reduce environmental pollution.

The stoichiometric equations involved in bioremediation when organic matter is removed are as follows (Wu et al., 2014):

4 $$0.0417{{\rm{C}}}_{6}{{\rm{H}}}_{12}{{\rm{O}}}_{6}+0.25{{\rm{H}}}_{2}{\rm{O}}=0.25{{\rm{CO}}}_{2}+{{\rm{H}}}^{+}+{{\rm{e}}}^{-}$$

5 $$0.25{{\rm{O}}}_{2}+{{\rm{H}}}^{+}+{{\rm{e}}}^{-}=0.5{{\rm{H}}}_{2}{\rm{O}}$$

6 $$0.0417{{\rm{C}}}_{6}{{\rm{H}}}_{12}{{\rm{O}}}_{6}+0.25{{\rm{O}}}_{2}=0.25{{\rm{CO}}}_{2}+0.25{{\rm{H}}}_{2}{\rm{O}}$$

Organic matter is represented by C_6_H_12_O_6_, which is oxidized to produce CO_2_, HCO_3_, and H_2_O. Molecular oxygen acts as the electron acceptor, and the CO_2_ produced is utilized by photoautotrophic microorganisms (cyanobacteria and diatoms) in periphyton communities for their growth. This process promotes the development of the periphytic community by creating a habitat for the attachment of other microorganisms (Wu et al., 2014).

## Physical and Chemical Conditions on River Biofilm Bioremediation

Environmental conditions significantly affect the species composition, microorganism growth, primary productivity, metabolism, and ecological function of periphyton. These conditions include temperature, pH, intensity of solar radiation, water flow, salinity, concentration of available nutrients, and oxygen levels (Tang et al., 2023).

The composition of microorganisms can be altered due to changes in water flow, which may cause some organisms to detach or add others to the biofilm. This factor facilitates the exchange of matter between the periphyton and the external environment (Shangguan et al., 2015). The temperature and intensity of solar radiation influence the biofilms’ growth. The temperature requirement for each type of microorganism can vary from 10 to 35 °C (Courtens et al., 2016). A high ratio of carbon to nitrogen (C/N) can lead to the proliferation of heterotrophic bacteria. In contrast, a low nitrogen to phosphorus (N/P < 16) ratio usually indicates nitrogen limitation, which can favor the growth of species capable of nitrogen fixation, such as certain cyanobacteria (Liepina-Leimane et al., 2024). Additionally, dissolved oxygen is a critical factor affecting the diversity and activity of bacteria, particularly those involved in the oxidation, nitrification, and denitrification of ammonia (Fitzgerald et al., 2015).

Physical factors can influence the bioremediation of contaminants by periphytic biofilms. One of the most critical factors is pH, as it impacts the growth of microorganisms and the activity of enzymes (Bhunia et al., 2022). Temperature also plays a crucial role, impacting periphyton’s growth and metabolism. Specifically, temperature influences the activity of enzymes, thereby affecting the metabolic rate of periphyton biota. Additionally, temperature plays a crucial role in determining the structure of the microbial community, especially for bacteria. Different dominant communities in periphyton exhibit distinct responses to temperature (Mu et al., 2020). Therefore, the optimal pH and temperature for the specific microorganisms forming the biofilm must be carefully optimized to achieve better results in bioremediation processes.

Nutrient availability also plays a vital role in the bioremediation of contaminants mediated by periphytic biofilms. Nitrogen is the most significant macronutrient for periphyton, making up about 10% of the dry mass of algae. The presence of nitrogen affects the structure of the microbial community. Studies indicate nitrogen is vital for periphyton growth and metabolism, but excessive nitrogen can hinder periphyton diversity. In addition to nitrogen, carbon and phosphorus also play essential roles in periphyton growth and community structure (Tang et al., 2023).

Sunlight, salinity, and contaminants’ physicochemical properties also impact bioremediation. Photosynthetic bacteria in periphyton require appropriate light radiation for their growth and, consequently, for contaminant removal. Light, either natural sunlight or artificial light, influences the rate of degradation by periphyton. Therefore, proper light radiation is crucial for effective periphyton growth and contaminant removal (Carles et al., 2021).

The impact of discharging effluent from effluent treatment stations (ETEs) on periphytic biofilms that form in these areas is a crucial point to consider. Several microorganisms can be carried from ETEs and thus cause effects on the microbial community of rivers and biofilms. These microorganisms can inhabit and alter the community’s composition, directly or indirectly, through species interactions. This can contribute to changes in respiratory profiles. Additionally, microbial communities released into wastewater should be viewed as a potential stressor for receiving streams, like other stressors such as nutrients, micropollutants, or increased temperature (Carles et al., 2022).

One way to improve the performance of natural biofilms in removing contaminants is through cell immobilization. Immobilizing biofilms on various substrates is a modern and efficient approach, as it enhances their activity and makes them more resilient to environmental disruptions (Chen et al., 2015; Shabbir et al., 2017b). Immobilization consists of adsorbing microorganisms onto a support matrix, such as gels, sponges, or porous materials, leading to the entrapment or incorporation of microorganisms in these materials. This process helps regulate the biomass’s shape, enhancing cell density, production rate, and product yield (Wu, 2017).

Shabbir et al. (2017a, 2018) demonstrated that immobilized biofilms (epiphyton, metaphyton, and epilithon) in bioreactors can completely decolorize and biodegrade both azo dyes and crystal violet. The process involves initial bioabsorption followed by biodegradation, converting dyes into non-toxic aliphatic compounds, highlighting the strong potential of immobilized periphyton for industrial-scale wastewater treatment.

## Current Challenges and Future Horizon

Growing concern for environmental sustainability has stimulated the search for efficient and low-impact remediation strategies. In this context, biofilm-based technologies show potential for environmental management, combining natural self-purification processes with applications in engineered systems. Although water purification performed by natural biofilms may appear limited when compared to conventional biological reactors due to lower biomass densities, biofilms are ubiquitous in fluvial systems, colonizing rocks, macrophytes, sediments, and artificial substrates. As a result, their cumulative contribution to river self-cleaning processes is substantial and warrants deeper investigation, both to elucidate underlying mechanisms and to optimize their performance.

A major challenge lies in the dynamic nature of biofilms. While they are effective in contaminant sorption and, in some cases, biodegradation, their structure and functionality are strongly influenced by hydrology, pollutant loads, nutrient availability, and climatic conditions. This variability directly affects system stability and makes performance prediction difficult, particularly under fluctuating environmental pressures. Moreover, most existing studies still focus on single-contaminant scenarios, which do not reflect the complex reality of aquatic environments. Investigations addressing multicomponent exposure scenarios are therefore essential to realistically assess the remediation potential of periphytic biofilms.

Non-biodegradable pollutants, such as heavy metals, pose a significant environmental challenge. These metals can be efficiently adsorbed or accumulated by the biofilm matrix and its associated microorganisms. However, the fate of metals following biofilm death or detachment raises critical concerns. When biofilms detach, the adsorbed metals may be released into the water column and sediments, potentially causing secondary contamination. In addition, non-biodegradable contaminants in rivers can enter the food chain when biofilms transfer these metals to other organisms. Strategies for the safe collection and disposal of metal-laden biofilms remain poorly developed, and their large-scale implementation continues to be a major obstacle.

Despite this, bioremediation using biofilms presents many advantages. In addition to directly removing contaminants, periphytic biofilms can also indirectly improve water quality by regulating nutrient availability and shaping microbial interactions. This ecological function extends their role beyond remediation, positioning biofilms as active regulators of ecosystem processes, particularly in systems affected by eutrophication and the proliferation of harmful cyanobacteria.

Future directions point to optimizing bioprocess design for both in situ applications, river self-cleaning capacity and engineered bioreactors. Advances in genetic engineering, enzyme technology, and ecological mapping may enhance biofilm resilience and expand the spectrum of degradable contaminants. A detailed study of the microbial communities that compose biofilms is essential, as their taxonomic and functional diversity determines bioremediation efficiency. Understanding which microorganisms and metabolic pathways are involved in degradation processes can help identify key species, predict ecosystem responses to environmental stressors, and guide the development of targeted interventions. Integrating microbial ecology with process engineering therefore represents a critical step toward unlocking the full potential of biofilms in environmental management, and it also highlights an important research gap in this field. Furthermore, strategies such as bioaugmentation, biostimulation, and cell immobilization can significantly improve biofilm activity and scalability.

Recent research has increasingly reflected global concerns about climate change, emphasizing its impacts on biofilms (Cantonati et al., 2024; Pacheco et al., 2022; Romero et al., 2018; Sentenac et al., 2023). This emerging field offers significant opportunities for investigation, and upcoming studies are expected to provide valuable insights in the coming years.

Looking ahead, river biofilm-based systems can be developed as nature-based solutions for integrated water management. Constructed and floating wetlands already demonstrate how biofilms can be leveraged in scalable projects, while advanced biofilm bioreactors can be used to treat industrial effluents containing persistent pollutants such as dyes, pharmaceuticals, and pesticides. Furthermore, periphytic biofilms can function as bioindicators of pollution, supporting environmental management in rivers and reservoirs. By framing these biofilms as self-sustaining microecosystems, future research can bridge fundamental understanding with practical applications, positioning periphytic biofilms as a pillar of environmental biotechnology and ecological engineering.

## Conclusion

Forming biofilms serves as a survival strategy for microorganisms, enabling them to withstand and tolerate harmful substances. Cells within a biofilm exhibit increased resistance to environmental fluctuations, making these structures particularly effective in detoxifying polluted environments. The interaction between biofilms and river water creates optimal conditions for natural attenuation processes, such as biodegradation and contaminant sorption. Notably, even at low contaminant concentrations, the adherence of biofilms to solid surfaces in rivers, both in sediments and in superficial areas, ensures a significant removal effect. As technological advancements progress, molecular studies on microbial diversity become increasingly feasible through the genomic analysis of microorganisms and their environments. This evolution paves the way for a deeper understanding of the structure and function of biofilm microbiomes in bioremediation and the establishment of genomic banks that can enhance the isolation of microorganisms and enzymes for biotechnological applications. Thus, research is being made to develop and identify contaminant removal techniques using periphytic biofilms, demonstrating their potential and offering new perspectives on their application.

## Data Availability

No datasets were generated or analysed during the current study.
